# The Role of MicroRNAs in Cardiac Stem Cells

**DOI:** 10.1155/2015/194894

**Published:** 2015-01-31

**Authors:** Nima Purvis, Andrew Bahn, Rajesh Katare

**Affiliations:** Department of Physiology-Heart Otago, Otago School of Medical Sciences, University of Otago, Dunedin 9010, New Zealand

## Abstract

Stem cells are considered as the next generation drug treatment in patients with cardiovascular disease who are resistant to conventional treatment. Among several stem cells used in the clinical setting, cardiac stem cells (CSCs) which reside in the myocardium and epicardium of the heart have been shown to be an effective option for the source of stem cells. In normal circumstances, CSCs primarily function as a cell store to replace the physiologically depleted cardiovascular cells, while under the diseased condition they have been shown to experimentally regenerate the diseased myocardium. In spite of their major functional role, molecular mechanisms regulating the CSCs proliferation and differentiation are still unknown. MicroRNAs (miRs) are small, noncoding RNA molecules that regulate gene expression at the posttranscriptional level. Recent studies have demonstrated the important role of miRs in regulating stem cell proliferation and differentiation, as well as other physiological and pathological processes related to stem cell function. This review summarises the current understanding of the role of miRs in CSCs. A deeper understanding of the mechanisms by which miRs regulate CSCs may lead to advances in the mode of stem cell therapies for the treatment of cardiovascular diseases.

## 1. Introduction

Stem cells are gaining interest as the treatment of choice in patients with cardiovascular disease who are resistant to conventional therapies. Stem cells are cell precursors which contribute to the formation of new tissues by a process involving cell differentiation, as well as a series of asymmetric divisions that lead to the production of daughter cells with different cell fates [1]. Recent studies demonstrate the superior effect of specialized stem cells such as cardiac stem cells (CSCs) in regenerating the diseased heart [2–8]. Although the difficulty of obtaining adequate cell numbers and invasiveness of the procedure necessary to obtain CSCs are recognized, these cells are preferred for stem cell therapy in patients with cardiovascular disease [6, 7]. Our recent studies suggest a key role of molecular signalling pathways in regulating the normal functions of cardiovascular cells [2, 9–11]. However, little is known about the molecular mechanisms that control the functions of CSCs. This review will provide a brief overview on the fundamental characteristics of CSCs and its subsets, followed by an in-depth analysis of the known physiological and pathophysiological roles of miRs as key molecular regulators in CSCs. Finally, the potential of modulating cardiac-specific miRs for therapeutic purposes will be discussed.

## 2. Cardiac Stem Cells

CSCs have recently acquired substantial clinical interest due to their ability to rapidly differentiate into functional cardiovascular cells. A pool of resident CSCs exist within the adult human myocardium and epicardium which are activated in response to ischaemic injury [12, 13]. Resident CSCs have the ability to differentiate into cardiomyocytes [14–17], smooth muscle cells [18], and endothelial cells [12, 19, 20], and in this way are able to maintain the physiological turnover of cardiac cells [21, 22]. CSCs can be isolated from myocardial or epicardial tissue and expanded* in vitro* to an appropriate number, so that they can be transplanted back* in vivo* in order to repair the damaged heart tissue [13, 15, 23, 24]. CSCs were first identified in the chick heart as early as 1943 [25], and since then, researchers have very well-characterized different subsets of CSCs in several species including mice and human. Mice and human CSCs are functionally identical in that they both differentiate into cardiomyocytes, although mice CSCs are known to grow at a slower rate than human CSCs* in vitro* [24, 26].

Another subgroup of stem cells identified within the myocardium are cardiac progenitor cells (CPCs). While both stem cell types exhibit similar markers and functional abilities, CSCs and CPCs can be distinguished on the basis of their progression through the process of myocardial differentiation. CSCs contain a higher proliferation and differentiation capacity, whereas CPCs are committed to differentiating into mature cardiomyocytes and have a limited capacity for self-renewal [27].

### 2.1. Subsets of CSCs

Several subsets of CSCs have been identified based on the expression of surface antigenic markers. These include stem cell antigen 1 (Sca-1), multidrug resistance protein 1 (MDR-1), c-kit protein, and islet1 (Isl1) [24]. Sca-1-positive CSCs form 70% of cells in the mouse heart after depletion of cardiomyocytes [28, 29]. They display a mesenchymal phenotype and are able to improve cardiac remodelling following myocardial infarction (MI) mainly by paracrine mechanisms [30]. Although the human ortholog of Sca-1 is yet to be identified, Smits et al. isolated Sca-1-like CSCs from the adult human heart using an anti-mouse Sca-1 antibody. These cells expressed early cardiac transcription factors (GATA-4, Mef2c, Isl1, and Nkx2.5) and differentiated into contractile cardiomyocytes [23]. Ryzhov et al. further demonstrated that Sca-1 like cells from the human heart also expressed mesenchymal stem cell markers CD105 and CD90 and confirmed the expression of cardiac specific genes when exposed to a cardiac differentiation medium [31].

The c-kit protein is commonly expressed on human CSCs and is thus used as the principle marker for the identification of CSCs in human heart tissue [24, 26]. Comparison of different CSC populations showed that c-kit-positive cells are the most primitive population in the heart [32]. However, following an injury, endogenous c-kit-positive CSCs migrate to the region of ischemic insult and differentiate into cardiomyocytes. Importantly, knockdown of endogenous c-kit-positive cells in the heart abolished the regeneration and functional recovery following experimentally induced myocardial damage in mice [33]. In addition, a number of preclinical studies [5, 33–36] and a recent clinical trial (SCIPIO) [37] demonstrated significant improvement in the regeneration of the diseased heart following transplantation of exogenous c-kit-positive cells into the myocardium. However, it should be noted that a recent study by van Berlo et al. showed very limited functional significance of c-kit-positive cells as the primary marker of CSCs [38], although further studies are required to confirm this notion.

Isl1 was initially hypothesized to be a specific marker for the second heart field (SHF) and to solely function in the development of SHF lineages. However, accumulating data strongly indicate that Isl1 is expressed in the common cardiac progenitor cell population and has an important function in heart development [39]. CSCs expressing Isl1 exhibited increased angiogenic gene expression and interestingly intramyocardial delivery of* isl1* gene markedly accelerated the functional recovery and reduced myocardial fibrosis in mice subjected to MI [40]. Bu et al. demonstrated the existence of primordial ISl1 progenitors in the human myocardium that give rise to multipotent ISl1 cardiovascular, lineages which are capable of differentiating into cardiomyocytes, smooth muscle cells, and endothelial cells when required [41].

### 2.2. Therapeutic Potential of CSCs

Autologous transplantation of CSCs into the diseased heart markedly improves its regeneration either through direct differentiation into the cardiovascular cells or more commonly through secretion of paracrine factors, which in turn activate regeneration processes.

A classic example for the differentiation effect of CSCs after transplantation was demonstrated by Beltrami et al. when they transplanted CSCs into an infarcted heart. Ten days after the transplantation of CSCs, they observed a thickened myocardium at the site of injection. CSCs, which were labelled with enhanced green fluorescent protein (EGFP) prior to injection, were found to replicate over time as evidenced by a substantially greater amount of fluorescence, indicating the existence of a large number of newly differentiated cardiomyocytes that originated from the transplanted CSCs [15].

Transplantation of CSCs also improved cardiac function in the human heart. Bolli et al. performed a randomised phase 1 trial (SCIPIO trial), in which 16 patients with postinfarction left ventricular dysfunction received autologous transfusion of c-kit-positive CSCs through coronary arteries. Fourteen out of 16 patients treated with CSCs showed a marked improvement in the left ventricle ejection fraction from 30.3% to 38.5% at four months after CSC transfusion, which further increased to 42% in 8 patients at the end of the 1-year follow-up period [42], thereby suggesting that CSCs could be an effective source for regeneration of the diseased heart. In the CADUCEUS trial, Makkar et al. demonstrated marked improvement in the viable myocardium in patients with MI who were treated with autologous cardiosphere-derived cells [43]. Although both these trials did not compare the effectiveness of other types of stem cells, results from similar studies suggest CSCs as a better option for cardiac regeneration compared to stem cell subtypes derived from other tissues. This is mainly because the CSCs are intrinsically programmed to form cardiomyocytes and increase cardiac tissue viability [20, 44–46]. In addition, resident CSCs are located nearer to the damaged site and reside in specialised niches in the myocardium, which specifically support their survival within the tissue [47]. Therefore, during stress/injury CSCs can migrate easily to the damaged site, where they rapidly proliferate and differentiate, allowing for a more accelerated and effective regeneration of the myocardium compared to other types of stem cells [29, 48].

In addition to these mechanisms, recently it was postulated that stem cells mainly exert their current beneficial effects via paracrine factors [49, 50] and several researchers have extensively studied various paracrine factors that could contribute to the protective effects of stem cells [51–53], including CSCs [54, 55]. Furthermore, a recent study by Chen et al. showed the ability of exosomes that are purified from the CPCs in protecting the mouse ischemic myocardium [56], suggesting a novel paracrine mechanism in CSC-induced protection.

## 3. MicroRNAs

MicroRNAs (miRs) are small (20–25 nucleotide), noncoding RNAs that regulate gene expression at the posttranscriptional level. They are involved in numerous cellular processes such as development, differentiation, and plasticity and have been recently found in various stem cell subtypes with varying modes of action such as stem cell fate and behavior [57]. Twenty four thousand miRs have been characterised over all species, each of which is predicted to target hundreds of messenger RNAs (mRNAs) [58, 59]. Most of the mammalian miRs are conserved across the species [60]. Nearly 2000 miRs have been identified in the human genome that regulate the expression of most human protein-coding genes [60]. Nevertheless, the physiological role of these miRs within stem cells is still relatively ambiguous.

### 3.1. Biogenesis

**Figure 1:**
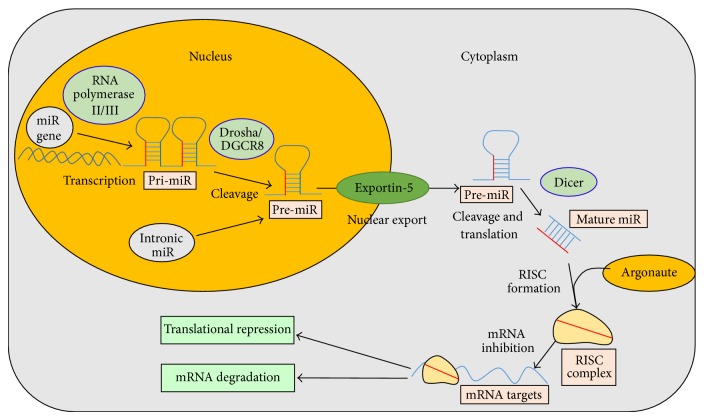
Schematic diagram illustrating the biogenesis of microRNAs (miRs). The diagram outlines this process from the initial transcription of miR genes or processing of intronic miRs in the nucleus until the formation of double-stranded, mature miRs in the cytoplasm, and their eventual inhibition/repression of target mRNA.

miRs can either be derived from genes or introns during splicing. Those derived from genes are initially transcribed in the nucleus by RNA polymerases (II and III) into primary transcripts called pri-miRs, which are processed into pre-miRs by a microprocessor complex consisting of the Drosha enzyme and RNA-binding protein, DiGeorge critical region 8 (DGCR8) [61]. The initial pri-miR precursors have long hair-pin structures with a terminal loop and flanking segments. Flanking segments are essential for the binding of DGCR8 to the pri-miR genes [62]. miRs derived from introns are called “mirtrons” and enter the miR biogenesis pathway by bypassing Drosha-mediated cleavage [63]. The pre-miRs are then transported through a nuclear pore into the cytoplasm by an exportin-5 transporter protein, where they are further processed into a double-stranded nucleotide intermediate by Dicer, an RNAse III enzyme. The intermediates are further processed into mature miRs by Dicer [57]. These mature miRs unwind within the complex, resulting in a single miR strand, which is finally incorporated, along with Argonaute (Arg) proteins, into an RNA-induced silencing (RISC) complex. The miR strand guides the RISC complex to conserved recognition sites on target mRNAs, where either translation is repressed by the miRs directly or the target mRNA is degraded. This largely depends on the complementary match of the miR to the target mRNA, which is mediated by the associated Arg proteins [64].  Figure 1 summarizes the biogenesis of miRs.

## 4. Physiological Role of miRs in CSCs

miRs are known to exist in a variety of different stem cells, where they act as molecular regulators of gene expression. The physiological roles of miRs are relatively well documented among neural stem cells, haematopoietic stem cells, or mesenchymal stem cells, although the physiological roles of miRs in CSCs have only been partially understood. This section will discuss the known physiological functions of miRs within CSCs.

### 4.1. miRs Regulate Myocardial Differentiation and Proliferation of CSCs

**Figure 2:**
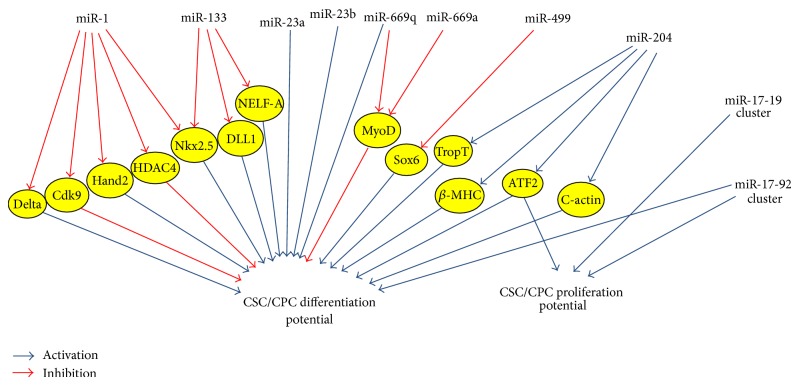
Summary diagram illustrating the cellular interactions of various miR subtypes and clusters with regard to their role of regulating the differentiation and proliferation potentials of cardiac stem cells (CSCs) and progenitor cells (CPCs). These miRs target specific transcription factors and cardiac genes (yellow circles) and inhibit their protein expression, causing a downstream effect on stem cell differentiation and proliferation (inhibition is indicated by the red arrows and activation is indicated by blue arrows).

Among the miRs expressed in the heart, miR-1 and miR-133 are the most commonly investigated miR subtypes, and importantly both have been demonstrated to be involved in regulating the differentiation of embryonic stem cells (ESCs). Reduced levels of miR-1 and miR-133 were observed in mouse ESCs following artificial induction of myocardial differentiation using trichostatin A, a histone deacetylase inhibitor. In conjunction with this, overexpression of miR-1 and miR-133 using lentiviral vectors reduced the expression of the cardiac specific gene, Nkx2.5 in ESCs (Figure 2) [65]. Furthermore, overexpression of miR-1 inhibited the translation of cdk9, a kinase which is known to activate cardiac-specific genes (Figure 2) [65]. The transcription factor, Hand2, was also identified as a target for miR-1 and interestingly Hand2 plays a major role in the formation of ventricular cardiomyocytes (Figure 2) [66]. These findings suggest that miRs within ESCs play a key role in the regulation of myocardial differentiation through inhibition of cardiac gene expression.

Available evidence suggests mixed results for the roles of miR-1 and miR-133 in regulating the functions of adult CSCs and CPCs [58, 66]. Ivey et al. demonstrated increased differentiation of CPCs by miR-1 by repressing the translation of DLL1, a transcription factor which promotes the expression of cardiac mesoderm genes and suppresses the expression of nonmesoderm genes (Figure 2) [58]. Conversely, miR-133 was found to inhibit this process and thus represses the differentiation of CPCs [58]. Although the molecular nature of this repression is unclear, the negative elongation factor-A (NELF-A), which is known to promote cardiogenesis, has been identified as a target for miR-133 [67]. Results from these studies show that the functional role of both the miRs in CSCs and CPCs largely depends on the nature of their molecular targets.

miR-1 and miR-499 were demonstrated to reduce proliferation and induce differentiation of human CPCs (72). miR-1 represses histone deacetylase 4 (HDAC4), a transcriptional inhibitor of muscle gene expression, while miR-499 represses sex determining region Y-box 6 (Sox6), a transcription factor involved in muscle differentiation (Figure 2) [68]. Moreover, when the activities of miR-1 and miR-499 were blocked using 2′-O-methyl oligonucleotides, differentiation of the CPCs was prevented [68]. This suggests that miR-1 and miR-499 expression are required for CPCs and gives greater supporting evidence for the general role of miRs in regulating the proliferation and differentiation of the CPCs, in which they reside.

The expression of miR-1 has also been discovered within CPCs of other species such as* Drosophila*, wherein miR-1 was shown to inhibit Notch signalling by targeting the translation of the Notch ligand, Delta* in vivo* (Figure 2) [69]. Notch signalling is known to inhibit cardiogenesis during ESC differentiation and therefore this finding correlates with a failure of CPCs to differentiate in miR-1 knockouts of* Drosophila* [69, 70]. Apart from the Delta ligand, miR-1 likely has many molecular targets that are important for cardiogenesis, although the overall mechanism is not fully understood [69].

Recent studies provide evidence for other less common miR subtypes such as miR-204, miR-669a, miR-669q, miR-23a, and miR-23b in promoting the differentiation of CPCs [71, 72]. Inhibition of miR-204 increased the proliferation of human CPCs, without affecting cell viability or apoptosis. This was associated with reduced differentiation potential and downregulation of cardiac-specific genes such as troponin T, *β*-myosin heavy chain, and cActin (Figure 2). The bioinformatics tool, GOmiR (Zotos, Athens, Greece), was used to identify activating transcription factor 2 (ATF2) as a target gene of miR-204 (Figure 2). This finding was significant, as overexpression of ATF2 in human CPCs elicited a similar increase in the proliferation potential of CPCs, thus revealing another molecular pathway that mediates the effect of miR-204 in enhancing the proliferation of CPCs [71].

The role of miR-669a and miR-669q was investigated by isolating neonatal CPCs from the *β*-sarcoglycan mutant mice which lack miR-669q and have reduced expression of miR-669a. When transplanted into infarcted hearts, these CPCs spontaneously differentiated into skeletal muscle fibres. However, overexpression of miR-669a and miR-669q in these CPCs reduced their differentiation potential into muscle fibres by inhibiting the expression of MyoD (Figure 2) [72].

In addition to the individual miRs, several miRs are also expressed in clusters. Individual members of each cluster are transcribed together to generate a polycistronic transcript [73]. The miR-17-92 cluster containing miR-17, -18, -19a, -19b, -20a, and -92a is expressed in CPCs during their regular development, where they are involved in increasing the proliferation of CPCs during development. This was confirmed through lentiviral mediated overexpression of the miR-17-92 cluster in the mouse heart. Overexpression of the miR-17-92 cluster resulted in a 2-fold increase in the proliferation rate of the CPCs [74]. These findings suggest that the proliferation capacity of endogenous CPCs can be regulated by miR clusters such as miR-17-92, suggesting a novel treatment modality in the field of stem cell therapy (Figure 2) [74]. Members of the miR-17-92 cluster family are regulated by a subtype of bone morphogenic protein, BMP4 as demonstrated by Wang et al., who showed that knocking down of BMP4 led to a reduction in the expression of the miR-17-92 cluster, as well as a delay in myocardial differentiation (Figure 2) [75]. In disease conditions such as diabetes, hypertension, hyperlipidaemia, myocardial differentiation and CSC proliferation are limited due to the decrease in the number of CSCs as a result of apoptosis [76]. However, as a consequence of their regulatory capability, miRs which induce differentiation and proliferation of CSCs, may be modulated to enhance this function in CSCs, although this hypothesis needs to be confirmed.

### 4.2. miRs Promote Neovascularisation and Angiogenic Differentiation of CSCs and CPCs

A recent study by van Mil et al. showed upregulation of miR-1 during angiogenic differentiation of human CPCs. In addition, overexpression of miR-1 enhanced vascular tube formation, spheroid sprouting, and migration of human CPCs through inhibition of antiangiogenic sprouty-related, EVH1 domain-containing protein 1 (spred1) protein [77]. These effects therefore provide evidence for the therapeutic effect of miR-1 in promoting angiogenesis in patients with ischemic heart disease or chronic degenerative diseases such as diabetes [2, 11]. miR-132 is another proangiogenic miR, which promotes neovascularisation in CPCs [78, 79]. We recently showed that saphenous vein-derived progenitor cells (SVPs) secrete miR-132 which, through inhibition of antiangiogenic protein p120RasGap, an inhibitor of proangiogenic VEGF, improved the vascular differentiation of SVPs. Transplantation of SVPs* in vivo* into the myocardium of mice subjected to MI markedly increased the expression of miR-132 and hence improved postischemic angiogenesis. In addition, secreted miR-132 also activated the endogenous CSCs, further promoting angiogenesis through vascular differentiation of activated CSCs. Importantly, knockdown of intracellular miR-132 resulted in the loss of proangiogenic potential of SVPs [4].

## 5. Pathophysiological Role of miRs in CSCs

**Figure 3:**
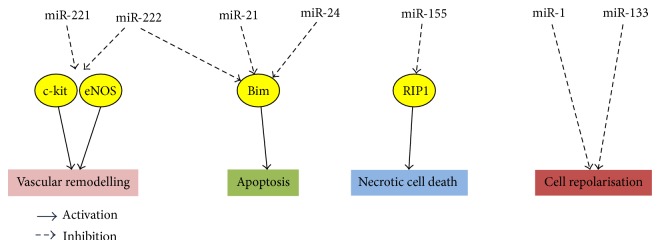
Pathophysiological effect of various miRs on vascular remodelling. Apoptosis, necrotic cell death, and potassium channel function. miRs within cardiac stem cells and progenitor cells regulate a number of known pathophysiological conditions. The effect of each miR subtype on these conditions as well as their associated target proteins is summarised in this diagram (inhibition is indicated by the dashed arrows and activation is indicated by solid arrows).

Accumulating evidence demonstrates that miRs are also involved and modulated in several pathologies, including cardiovascular pathology. Therefore, a better understanding of the underlying mechanisms through which miRs affect disease processes is vital for the development of stem cell therapies. The known effect of each miR subtype on the different pathologies is summarised in Figure 3.

### 5.1. miRs in CSCs Interfere with Vascular Remodelling

Vascular remodelling is an active process characterised by alterations in the structure and thickness of the luminal walls of blood vessels, which causes a change in systemic vascular resistance and thus blood pressure [80]. A series of studies demonstrated the impact of miRs in influencing vascular remodelling. Albinsson et al. showed thinning of the blood vessel wall due to decreased proliferation of vascular smooth muscle cells following deletion of dicer, the enzyme essential for the maturation of miRs. Furthermore, smooth muscle cells in Dicer knock-out mice showed a reduction in the medial thickness of the aorta as a result of the loss of actin stress fibres, further validating the effect of miRs in vascular development [81].

miR-221 and miR-222 were both demonstrated to inhibit endothelial cell migration and proliferation and vascular remodelling* in vitro* by targeting c-kit, as well as indirectly reducing the expression level of endothelial nitric oxide synthase expression (eNOS, Figure 3) [82]. In haematopoietic progenitor cells, miR-221 and miR-222 reduced c-kit expression leading to reduced cell proliferation [83]. The mechanism involving c-kit inhibition is unclear; however c-kit being an important marker for CSCs, it can be speculated that miR-221 and miR-222 are also involved in CSC differentiation and function [26]. A recent study showed a crucial role for eNOS in the mobilization and functional activity of stem and progenitor cells [84]. This evidence indicates that certain miRs could interfere with vascular remodelling and hence should be avoided in the future during the selection of miRs to improve the efficiency of stem cell therapy. Furthermore, associated chronic diseases such as diabetes or hyperlipidemia can also affect the pathophysiological roles of miRs in CSCs. For example, diabetes upregulates both miR-221 [85] and miR-222 [86]; hence it is plausible that these miRs are also upregulated in CSCs from the diabetic heart. Thus, inhibiting the expression of miR-221 and miR-222 in CSCs may be beneficial in preventing the development of vascular dysfunction in people with diabetes.

### 5.2. miRs in CSCs and CPCs Inhibit Apoptosis and Necrotic Cell Death

Recent studies suggest the involvement of CSC-derived miRs in cell death pathways such as apoptosis. For instance, treatment of CPCs with a cocktail of miR-21, -24, and -221 was able to inhibit the critical apoptotic activator B-cell lymphoma 2 interacting mediator of cell death (Bim) and therefore improve the survival of CPCs (Figure 3) [87]. Moreover,* in vivo* transplantation of cocktail-treated CPCs into an infarcted myocardium markedly improved the engraftment of transplanted CPCs, as evidenced by a longer survival rate and increased myocardial wall thickness [87]. miR-125a, a member of the let-7 family, is also highly expressed in CSCs and has a major role in regulating stem cell apoptosis, through which it contributes to the maintenance of the CSC store [88].

In addition to apoptosis, miRs are also shown to have an effect on the necrotic cell death of CSCs. Liu et al. demonstrated a 4-fold increase in miR-155 expression in CSCs when the cells are exposed to prooxidant hydrogen-peroxide [89]. Exposure of pre-miR-155 treated CSCs to oxidative stress consistently repressed necrotic cell death of CSCs by targeting the receptor interacting protein 1 (RIP1), a protein required for activation of necrosis (Figure 3) [89].

Apoptosis and necrotic cell death are the major causes for reduction in the number of CSCs following* in vivo* transplantation and in chronic morbidities including diabetes [2, 10, 90]. Hence, it is tempting to speculate that miRs such as miR-221, -21, -24, -125, and -155 that inhibit apoptotic and necrotic cell death pathways may be extremely important to improve the efficacy of stem cell therapy in these patients.

### 5.3. miRs in CPCs Promote Dysfunctional Potassium Channels

The potassium channels, KCNE1 and KCNQ1, have been identified in CPCs, where they aid in maintaining cell repolarisation [91]. Abnormal expression of miR-1 and -133 was demonstrated to provoke cardiac arrhythmias by altering the potassium current in cardiomyocytes [92]. Consistently, exposure of CPCs to high glucose augmented the expression of miR-1 and miR-133 along with suppression of KCNE1 and KCNQ1 (Figure 3). Of note, treatment of CPCs with anti-miR-1 and -133 restored the function of potassium channels [91]. This finding suggests that miR-1 and miR-133 are involved in regulating potassium channels within CPCs and that this function may be impaired in cardiovascular pathologies.

## 6. Therapeutic Use of miRs in CSCs

As indicated in the previous sections, due to their physiological and pathophysiological role in CSCs, miRs have the potential to be modulated to improve CSC function and its efficacy in various cardiovascular diseases. Moreover, the primary role of miRs in promoting the differentiation and proliferation potential of stem cells enables their use in a number of other therapeutic settings.

Although limited, emerging evidence strongly supports the major role of miRs in CSC-based therapies. Inhibition of miR-378 was demonstrated to indirectly promote CSC viability by enhancing the expression of connective tissue growth factor (CTGF). CTGF functions as an autocrine growth factor and is known to exert its effect on tissue repair, scarring, fibrosis [93], cytoprotection against ischaemic reperfusion injury [94], and endothelial cell adhesion and survival [95]. Electrical stimulation of CSCs augmented the expression of CTGF through downregulation of miR-378, suggesting that inhibition of miR-378 could be used to improve the survival of CSCs [96].

miR-499 has been recently shown to have an important role in influencing the cardiomyocyte differentiation of CSCs. Under normal conditions, miR-499 expression is negligible in CSCs, although cardiomyocytes express high levels of miR-499. However, when required, miR-499 traverses gap junction channels and translocates to structurally coupled CSCs, favouring their differentiation into functionally competent cells through repression of miR-499 target genes, Sox6 and regulator of differentiation 1 (Rod1) (Figure 2). Furthermore, transplantation of miR-499 expressing CSCs into infarcted rat hearts accelerated the development of new cardiomyocytes, eventually leading to the improvement of cardiac function [97].

In a very recent study, Izarra et al. demonstrated the ability of miR-133 in improving the protective ability of CPCs. They demonstrated a marked increase in miR-133 expression in CPCs exposed to differentiation medium. Further, CPCs overexpressed with miR-133 showed a marked reduction in caspase activity through inhibition of proapoptotic Bim.* In vivo* transplantation of miR-133 overexpressing CPCs consistently demonstrated superior recovery of cardiac functions [98].

The application of miRs to CSCs to treat myocardial infarction is a newly emerging treatment strategy and one that has gathered much more evidence for its therapeutic effects in recent years. Despite a lack of documentation for this treatment method, specific miRs such as miR-378, miR-499, and miR-133 have been shown to effectively regenerate myocardial tissue in animal models. However, it is apparent that more research is needed to fully understand whether the capacity of the miRs to regenerate the myocardium can be translated to clinical practice and whether this is as effective in humans as in animal models.

## 7. Concluding Remarks

A number of miR subtypes have been investigated for their role in regulating the differentiation and proliferation of CSCs. However, several questions still remain unanswered such as if the effect of miRs will depend on age, gender, associated diseases, treatments, ethnicity, and environmental factors. Therefore, future studies focusing on answering these important questions will provide novel insights into the pathophysiological role of miRs in CSCs, which eventually result in the development of new therapeutic modalities. Based on the experimental evidence described within this review, a general consensus can be drawn.

1. The primary function of miRs within CSCs involves regulating the differentiation and proliferation potential of these cells.
2. miRs within CSCs are involved in a number of physiological functions, mostly based on their ability to target the mRNA of transcription factors and other regulatory molecules.
3. miRs within CSCs have implications in pathological settings, as they are responsible for influencing the progression of a number of pathological processes such as apoptosis and necrosis.
4. Modulation of cardiac-specific miRs has the potential to be an effective strategy in CSC therapies for the purposes of treating cardiac-related diseases. The therapeutic potential of miRs has been indicated from an experimental standpoint in animal models. However, whether the same can be replicated in humans and most importantly, whether one or a cocktail of miRs are necessary to produce the desired clinical effect, is yet to be investigated.
